# From feedback orientation to academic emotions: the indirect association through agentic engagement among secondary vocational students

**DOI:** 10.3389/fpsyg.2026.1959575

**Published:** 2026-10-19

**Authors:** Juan Gao, Lan Yang

**Affiliations:** 1 School of Educational Science, Hunan University of Arts and Science, Changde, China; 2 Department of Curriculum and Instruction, The Education University of Hong Kong, Tai Po, Hong Kong SAR, China

**Keywords:** academic emotions, agentic engagement, educational assessment, educational psychology, feedback orientation, self-determination theory, vocational education

## Abstract

Feedback is integral to vocational learning, yet receiving feedback does not ensure that students value it, feel capable of using it, or actively contribute to the feedback process. This gap is particularly relevant in secondary vocational education, where learning often involves repeated practice, explicit performance standards, and structured professional preparation. Drawing on a Self-Determination Theory-informed perspective, this study examined how feedback orientation is associated with agentic engagement and positive academic emotions among 452 students from a Chinese secondary vocational school. Confirmatory factor analysis supported feedback orientation as a multidimensional but strongly integrated construct comprising feedback utility, self-efficacy, social awareness, and accountability. Structural equation modeling showed that feedback orientation was positively associated with agentic engagement (*β* = 0.662), which was in turn positively associated with positive academic emotions (*β* = 0.482). A significant indirect association was also observed (*β* = 0.319), alongside a remaining direct association between feedback orientation and positive academic emotions. These findings extend feedback-orientation research from how students perceive feedback to how these orientations are associated with proactive participation and emotional experience. They highlight agentic engagement as one plausible component of this relationship rather than a sole or causal mechanism. Given the cross-sectional, single-school, predominantly female, self-report design, longitudinal, multi-method, and multi-site research is needed to establish temporal processes and examine the generalizability of these relationships across vocational contexts.

## Introduction

Secondary vocational education (SVE) is an important upper-secondary pathway in China. It includes secondary specialized schools, skilled-workers’ schools, and vocational upper-secondary schools, and is intended to develop practical skills relevant to occupational and labor-market demands (Wu et al., 2025). National evidence also indicates that SVE enrolment differs from academically oriented upper-secondary education in students’ socioeconomic and gender composition (Wang and Guo, 2019). Within the technical and skills-based programs examined in the present study, learning combines disciplinary knowledge with repeated practice of occupation-specific procedures and performance standards relevant to future professional work. Feedback is consequently often connected to observable task execution, the quality of performance, and students’ progress toward expected standards.

Frequent corrective information, however, does not automatically become learning. Its educational value depends substantially on whether students interpret feedback, judge its relevance to their work, and use it to guide subsequent action (Carless, 2022; Hattie and Gan, 2011). This distinction is especially consequential in secondary vocational education: feedback may be experienced simply as correction to comply with, or as information that students actively use to improve developing competence. The present study therefore examines students’ feedback orientation as a learner characteristic that may help explain variation in how they participate in feedback processes and experience learning emotionally.

This learner-centered view of feedback is particularly relevant in vocational education. Feedback can support students’ developing professional competence when it helps them understand performance standards and identify feasible next steps. Yet it may be experienced merely as correction to be followed when students do not understand its purpose, lack confidence in using it, or perceive limited scope to discuss and act on it. Research on feedback in vocational learning similarly highlights its role in students’ continuing development as learners and emerging practitioners rather than as passive recipients of evaluative information (Johansson, 2020). More generally, reviews of feedback interventions show that feedback effects are contingent on how feedback processes are designed, interpreted, and enacted by learners (Ajjawi et al., 2022; Winstone et al., 2017).

Feedback orientation offers a useful framework for examining these differences in students’ engagement with feedback. Originally developed in organizational research, feedback orientation refers to an individual’s general receptivity to feedback, including perceived feedback utility, feedback self-efficacy, feedback social awareness, and feedback accountability (Linderbaum and Levy, 2010). In educational settings, these dimensions concern whether students recognize feedback as useful for improvement, believe that they can understand and apply it, appreciate its interpersonal implications, and accept responsibility for responding to it (Yang et al., 2014). Thus, feedback orientation concerns not the amount or quality of feedback provided by teachers, but students’ dispositions toward making use of feedback.

Evidence in educational contexts indicates that these dispositions matter. Yang and Yang (2018) found that feedback beliefs were associated with student engagement in Chinese secondary-school settings, including vocational education. Yang (2022) further argued that feedback orientation may help learners transform externally provided information into opportunities for subsequent learning. Complementing this educational evidence, Katz et al.’s (2023) meta-analysis of 46 workplace samples (*N* = 12,478) found that feedback orientation was positively associated with learning-goal orientation (*r* = 0.39), performance (*r* = 0.35), and feedback seeking (*r* = 0.43). These workplace findings should not be generalized directly to secondary vocational students; nevertheless, they provide convergent evidence that receptivity to feedback is associated with learning-related and performance-related behavior.

Self-Determination Theory (SDT) provides a useful motivational lens for interpreting these feedback-related dispositions. SDT proposes that autonomy, competence, and relatedness are basic psychological needs that shape the quality of motivation, engagement, and well-being (Ryan and Deci, 2000, 2017). Feedback may be more readily used when students perceive it as relevant to valued learning goals, feel capable of understanding and applying it, and experience feedback interactions as respectful and supportive. From this perspective, feedback can support learning not simply because it identifies a performance discrepancy, but because students experience it in ways that permit competent, self-endorsed, and relationally secure participation.

The four dimensions of feedback orientation can be interpreted within this SDT-informed account, while remaining conceptually distinct from direct measures of basic psychological need satisfaction or motivational regulation. Feedback utility reflects whether students perceive feedback as valuable for learning and improvement; feedback self-efficacy reflects their perceived capacity to interpret and use it; and feedback social awareness reflects the interpersonal meaning of feedback exchanges (Linderbaum and Levy, 2010; Yang et al., 2014). Feedback accountability concerns students’ willingness to assume responsibility for responding to feedback and improving their learning. In educational feedback processes, such responsibility is important because learners need to make judgments and take action rather than simply receive feedback (Carless, 2022; Yang, 2022). Accountability is consistent with the SDT concept of autonomy when students personally endorse responsibility for acting on feedback and experience this action as self-directed (Reeve et al., 2012; Ryan and Deci, 2017). However, it should not be treated as equivalent to intrinsic motivation or autonomous regulation. Students may also act on feedback because of external expectations, fear of negative evaluation, or compliance with authority. Because the present study does not assess students’ reasons for responding to feedback, accountability is interpreted as a feedback-related disposition rather than direct evidence of autonomous motivation.

A central question, therefore, is how feedback orientation may be associated with students’ active participation in instruction. Agentic engagement is especially relevant because it captures students’ constructive contributions to the learning process, such as asking questions, expressing preferences or difficulties, requesting clarification, and seeking more useful learning support (Reeve, 2013; Reeve and Tseng, 2011). These actions may be particularly important in feedback contexts, where students often need clarification, elaboration, or opportunities to discuss how feedback applies to their work. Recent conceptual work also emphasizes that agentic engagement involves students’ attempts to influence the instruction and support they receive, rather than simply complying with teacher direction (Reeve and Jang, 2022).

A feedback-oriented student may consequently be more inclined to ask for clarification, communicate learning needs, and seek feedback that can be acted upon. Nevertheless, agentic engagement should not be assumed to be uniformly beneficial or to represent the only process linking feedback orientation with learning outcomes. The meaning and consequences of agentic action depend partly on teacher responsiveness and the wider instructional environment. When students’ questions and contributions are acknowledged, agentic engagement may facilitate clearer understanding and more productive feedback use. When initiative is ignored or discouraged, the same efforts may not yield supportive learning experiences. The present study therefore examines agentic engagement as one theoretically relevant concurrent correlate, rather than as a comprehensive explanation of how feedback orientation operates.

Academic emotions provide an important outcome domain for this inquiry. Emotions such as enjoyment, hope, anxiety, frustration, and boredom shape students’ attention, persistence, use of learning strategies, and engagement (Pekrun and Linnenbrink-Garcia, 2012). Feedback is emotionally consequential because students may appraise it as informative and manageable, or as threatening, unclear, and difficult to use. Research has shown that anticipated feedback can influence achievement-related emotions, partly through students’ appraisals of control and value (Pekrun et al., 2014). Studies of feedback orientation in higher education likewise indicate associations between students’ orientations toward feedback and learning-related emotions, although evidence remains limited in secondary vocational education (Frondozo and Yang, 2023; Yang et al., 2023c).

Three gaps therefore motivate the present study. First, evidence remains limited on the concurrent association between feedback orientation and academic emotions in secondary vocational education. Second, agentic engagement has rarely been examined as a feedback-relevant form of student participation in this context. Third, the psychometric structure of the 12-item short-form Feedback Orientation Scale requires further examination among Chinese secondary vocational students. Accordingly, this study evaluates the scale’s factor structure and examines whether agentic engagement statistically accounts for part of the concurrent association between overall feedback orientation and academic emotions.

## Literature review

### Feedback orientation as a motivational disposition within self-determination theory

Self-Determination Theory (SDT) explains how students’ motivation and psychological functioning develop through continuing interactions between learners and their social environments. Its central proposition is that the internalization of externally prompted activities and the maintenance of intrinsic motivation depend substantially on the satisfaction of three basic psychological needs: autonomy, competence, and relatedness (Deci and Ryan, 2012a; Evans, 2015). Autonomy refers to experiencing one’s actions as volitional and self-endorsed, competence concerns feeling capable of meeting environmental demands, and relatedness involves feeling connected to and valued by significant others. These needs are not satisfied independently of context. Rather, their satisfaction is shaped by the interpersonal and instructional cues that students encounter during learning (Chen et al., 2015; Deci and Ryan, 2012b; Ryan and Deci, 2017).

Feedback constitutes one such consequential instructional cue because it communicates information about students’ current performance, future possibilities, relationships with others, and responsibility for improvement (Tanes et al., 2011; Yang et al., 2023a). Its motivational significance, however, cannot be understood solely in terms of the information delivered. Students differ in how they perceive, interpret, and respond to feedback, and these differences are captured by the construct of feedback orientation. Feedback orientation refers to individuals’ general dispositions toward feedback and comprises four related dimensions: utility, self-efficacy, social awareness, and accountability (Yang, 2022). Utility concerns the perceived value of feedback for learning and improvement; self-efficacy refers to confidence in understanding and acting on feedback; social awareness captures sensitivity to the interpersonal purposes and consequences of feedback; and accountability reflects a willingness to accept responsibility for responding to feedback. These dimensional definitions follow the original Feedback Orientation Scale and its educational adaptation (Linderbaum and Levy, 2010; Yang et al., 2014).

From an SDT perspective, feedback orientation can be conceptualized as a motivational disposition that influences whether students approach feedback as a resource for growth or as an externally imposed judgment. Students who perceive feedback as useful are more likely to recognize its personal relevance and to internalize the reasons for engaging with it. This process is consistent with SDT’s account of internalization, whereby externally initiated activities become increasingly self-endorsed when learners understand and accept their value (Deci and Ryan, 2013, 2016). Autonomy-supportive feedback may therefore strengthen students’ willingness to engage with feedback because it communicates meaningful rationales, acknowledges students’ perspectives, and preserves a sense of volition. Evidence that autonomy-supportive feedback is associated with more autonomous motivation supports this interpretation (Carpentier and Mageau, 2016; Deci and Ryan, 2013).

Feedback self-efficacy is similarly relevant to the need for competence. Competence satisfaction develops when students experience themselves as capable of understanding expectations, meeting challenges, and using available resources effectively. It therefore involves not only possessing skills but also having opportunities to apply them successfully (Kumar and Kaur, 2019). Feedback that is clear, actionable, and appropriately challenging may strengthen students’ confidence in their ability to improve, whereas vague or controlling feedback may reinforce uncertainty and perceived incompetence. Personalized feedback may also support competence indirectly by strengthening students’ sense of relatedness, which can enhance self-efficacy and the effort invested in learning (Ajjawi et al., 2022).

The social awareness dimension of feedback orientation is closely connected to relatedness because feedback is embedded in interpersonal relationships rather than transmitted in a social vacuum. Relatedness refers to feeling socially connected, cared for, and able to contribute meaningfully within a group (Ryan and Deci, 2017). Students who are sensitive to the interpersonal functions of feedback may be more likely to recognize teachers’ intentions, maintain constructive feedback relationships, and respond in ways that preserve mutual trust. Teachers can support this process by explaining the rationale for instructional decisions and communicating their intentions transparently, thereby helping students interpret feedback interactions more accurately and strengthening teacher–student relationships (Wang et al., 2025). In contrast, weak social connection or an unsupportive feedback climate may frustrate relatedness and contribute to reduced motivation, diminished responsibility, and psychological distress (Ryan and Deci, 2000).

Feedback accountability must be distinguished from autonomous internalization. The same observable act of revising work can arise from identified regulation, in which the student accepts the value of improvement, or from controlled regulation, in which the student responds to surveillance, authority, guilt, or fear of disapproval (Deci and Ryan, 2016; Reeve et al., 2012). Hierarchical vocational classrooms may make both forms plausible. The present measure assesses perceived responsibility to act on feedback but does not assess why that responsibility is accepted; accountability therefore cannot be equated with autonomy in the present data.

This distinction is especially important in highly structured vocational settings, where repeated routines and externally specified performance standards may support skill acquisition but may also narrow students’ experience of volition. Recent SDT-related work has proposed novelty as an additional experiential resource associated with psychological well-being, including in structured physical-education contexts (González-Cutre et al., 2016; Trigueros and García-Mas, 2025). These findings do not establish novelty as a mechanism in the present vocational-feedback model, but they highlight that motivational quality may depend on whether structured learning still provides opportunities for exploration, meaningful choice, and personally endorsed action. Relatedly, Franco et al. (2026), in a university mindfulness intervention, reported changes in motivation, learning strategies, procrastination, and self-regulation. Although that context differs from secondary vocational education, it reinforces the broader proposition that a favorable feedback orientation may need to operate alongside self-regulatory resources if students are to process feedback autonomously rather than merely comply with external correction.

These four dimensions should not be interpreted as direct equivalents of the three basic psychological needs. Rather, SDT provides a motivational explanation for why feedback orientation may develop and why it matters for subsequent learning. Need-supportive feedback environments may encourage students to perceive feedback as useful, manageable, relationally constructive, and worthy of personal responsibility. In turn, stronger feedback orientation may increase students’ readiness to engage actively with feedback. Theoretically, feedback orientation therefore represents an important link between the social conditions surrounding feedback and students’ proactive participation in learning.

The educational articulation is supported most directly by Yang and Yang (2018), who studied vocational and mainstream secondary students in mainland China. Their model treated utility, self-efficacy, social awareness, and accountability as students’ feedback-related self-processes and found that utility, self-efficacy, and accountability were positively associated with engagement across both samples. This evidence supports the relevance of feedback beliefs to participation while leaving open whether accountability was autonomously endorsed or socially controlled.

### Agentic engagement as students’ proactive contribution to feedback processes

Student engagement has traditionally been conceptualized in behavioral, emotional, and cognitive terms. Agentic engagement extends this framework by capturing students’ intentional and constructive contribution to the flow of instruction (Reeve and Tseng, 2011; Reeve, 2013). It is not merely a general form of proactive behavior, nor is it interchangeable with participation or effort. Rather, agentic engagement refers to the ways in which students attempt to enrich, personalize, or redirect instruction so that it becomes more supportive of their learning needs, interests, and goals. Agentically engaged students may ask questions, communicate preferences, request clarification, express learning needs, offer suggestions, or seek additional resources. These actions are proactive, purposeful, collaborative, and constructive because they are directed toward improving the quality of the learning experience rather than simply complying with existing instructional demands (Reeve, 2013; Patall, 2024).

This conception is especially relevant to feedback. Contemporary feedback research increasingly recognizes that learners are not passive recipients of information but active participants who help determine whether feedback becomes educationally useful (Cook-Sather et al., 2021; Price et al., 2011). Feedback has limited value when students merely receive comments without seeking clarification, evaluating their relevance, or using them to regulate subsequent action. By contrast, students who engage agentically can influence the feedback process by asking for more specific information, communicating confusion, requesting examples, negotiating goals, and identifying the support they need. In this sense, agentic engagement provides a behavioral expression of students’ willingness to participate actively in feedback processes. The examples of clarification seeking and expressed learning needs are consistent with agentic-engagement and proactive-recipience accounts (Reeve, 2013; Reeve and Jang, 2022; Winstone et al., 2017).

Winstone et al. (2017) conceptualized this active role as “proactive recipience,” emphasizing that learners share responsibility for generating, interpreting, and using feedback. Proactive recipience is conceptually compatible with agentic engagement because both position students as contributors to, rather than merely recipients of, instructional processes. Yang and Yang (2018) similarly showed that students’ willingness to assume responsibility for feedback is associated with stronger engagement in feedback-based learning. Feedback orientation may therefore function as a motivational antecedent of agentic engagement. Students who value feedback, feel capable of using it, understand its relational implications, and accept responsibility for improvement should be more likely to initiate and sustain constructive feedback interactions.

From Reeve’s perspective (e.g., Reeve, 2013; Reeve and Jang, 2022), agentic engagement is reciprocal: supportive instruction can encourage student initiative, while students’ contributions can provide teachers with information for adjusting explanations, pacing, feedback, and task structures. This reciprocity cannot be assumed in a rigid environment. When teachers respond constructively, students’ questions and expressed needs may improve instructional fit. When initiative is ignored, discouraged, or treated as noncompliance, the same behavior may fail to alter instruction and may be followed by frustration, anxiety, superficial compliance, or withdrawal. Agentic engagement is therefore examined as a plausible, context-dependent intermediary rather than a mechanism that necessarily improves emotions.

This reciprocal process is central to SDT. Basic psychological need satisfaction and self-efficacy have been identified as motivational antecedents of agentic engagement because students are more likely to contribute proactively when they feel volitional, capable, and relationally secure (Dostmohamadi et al., 2021; Sökmen, 2021). At the same time, agentic engagement may further support these needs by enabling students to obtain clearer guidance, more appropriate challenges, and more responsive interpersonal support. It therefore operates as both an outcome of motivationally supportive conditions and a means through which students help create more supportive learning experiences.

Despite its theoretical importance, agentic engagement remains less frequently examined in feedback research than behavioral, cognitive, or emotional engagement. Existing work indicates that students’ active engagement is relevant to how they interpret and use feedback (Almusharraf and Bailey, 2021; Winstone et al., 2017), but evidence is limited on its concurrent association with feedback orientation and emotional experience in vocational education. The present study therefore treats agentic engagement as one theoretically plausible intermediary variable within the specified statistical model. It does not assume that feedback orientation automatically produces emotional benefits or that agentic engagement is the only relevant process.

### Academic emotions as psychological outcomes of feedback-related engagement

Psychological outcomes in education encompass students’ cognitive appraisals, emotional experiences, motivational states, and behavioral tendencies during learning (Anderman, 2002; Reeve, 2024). The present study focuses specifically on academic emotions because they represent an important indicator of how students experience and adapt to instructional demands. Academic emotions are emotions directly related to learning activities, achievement tasks, or academic outcomes. They include positive experiences such as enjoyment, hope, and pride, as well as negative experiences such as anxiety and boredom (Pekrun and Linnenbrink-Garcia, 2012; Ullrich-French et al., 2012). These emotions are educationally consequential because they influence attention, persistence, strategy use, self-regulation, and willingness to remain engaged with challenging learning activities (Pekrun and Linnenbrink-Garcia, 2012).

Feedback is a particularly salient context for academic emotions because it provides students with evaluative information about their competence, progress, and standing in relation to expectations. Students may experience hope or pride when feedback confirms progress and communicates attainable pathways for improvement. They may also experience anxiety, shame, frustration, or boredom when feedback is perceived as threatening, unclear, controlling, or irrelevant. Emotional reactions can occur immediately following feedback and may subsequently influence students’ academic behavior and performance (Inventado et al., 2011). Feedback emotions should therefore not be treated as incidental reactions; they are part of the psychological process through which students interpret and respond to instructional information.

The SDT offers a useful explanation for variation in these emotional experiences. When learning environments support autonomy, competence, and relatedness, students are more likely to experience autonomous motivation, psychological vitality, and positive emotions (Deci and Ryan, 2012a, 2014). Autonomy support allows students to experience learning as self-endorsed rather than coerced. Competence support helps students interpret challenges as manageable and improvement as possible. Relatedness support provides the interpersonal security needed to receive and discuss feedback without excessive defensiveness or threat. Conversely, frustration of these needs may contribute to negative emotional experiences. Autonomy frustration, perceived incompetence, and relational disconnection have each been associated with maladaptive emotions, including anxiety and other forms of psychological distress (Deci and Ryan, 2013; Howard et al., 2025).

Autonomous motivation may also enable students to process difficult or unfavorable experiences more openly. Weinstein and Hodgins (2009) suggested that autonomy facilitates less defensive and more integrative processing of negative emotional information. Applied to feedback, students who experience greater autonomy may be better able to consider critical comments without interpreting them solely as threats to self-worth. In a similar manner, feedback environments that support students’ psychological needs may foster more adaptive emotional responses, whereas controlling or unsupportive environments may intensify negative emotions (Akolgo et al., 2025).

However, students’ emotions are not determined only by what teachers provide. Reeve’s account of agentic engagement highlights students’ capacity to influence the motivational quality of their own learning environments. A student who asks for clarification may reduce uncertainty; a student who requests a more manageable example may strengthen competence; and a student who communicates a preference or concern may increase autonomy and relational understanding. Through these contributions, agentic engagement may help students obtain feedback that is more comprehensible, responsive, and useful. Such experiences may, in turn, be associated with more positive academic emotions and fewer maladaptive emotional reactions. This interpretation follows Reeve’s account of students as active contributors to motivationally supportive instruction (Reeve, 2013; Reeve and Jang, 2022).

This reasoning supports a conditional indirect association rather than a uniformly positive sequence. Students with stronger feedback orientation may report more questioning, clarification seeking, and expression of learning needs. These actions may coincide with more positive emotions when the environment is responsive, but they may coincide with maladaptive emotions when the environment remains controlling or unresponsive. Because teacher responsiveness and the full spectrum of discrete emotions were not measured, the present analysis tests only whether the aggregate variables are concurrently associated in the proposed pattern.

Accordingly, agentic engagement was selected because it is the engagement dimension most directly concerned with students’ attempts to influence instruction and feedback dialog. It was not selected because alternative processes are theoretically unimportant. Feedback appraisal, emotion regulation, teacher responsiveness, and other forms of engagement may also account for or condition the association between feedback orientation and academic emotions, but they were outside the available data.

In summary, the study tests a parsimonious SDT-informed model of concurrent associations. Feedback orientation represents students’ dispositions toward feedback; agentic engagement represents reported proactive contribution; and academic emotions represent their overall emotional experience. The model does not establish that feedback orientation temporally precedes engagement, that engagement causes emotional change, or that proactive action is beneficial regardless of context. The three constructs are linked by prior work on feedback beliefs, agentic participation, and achievement emotions, but the proposed ordering remains a statistical specification (Pekrun et al., 2014; Reeve, 2013; Yang and Yang, 2018).

### Current study and research aims

Guided by Self-Determination Theory and previous evidence linking students’ feedback beliefs with engagement (Yang and Yang, 2018), agentic engagement with students’ constructive influence on instruction (Reeve, 2013), and feedback appraisal with academic emotions (Pekrun et al., 2014), this study pursued three related aims among secondary vocational students:

To evaluate alternative factor-structural representations of the 12-item short-form Feedback Orientation Scale.

To examine the concurrent associations of overall feedback orientation with agentic engagement and academic emotions.

To test whether agentic engagement statistically accounted for an indirect association between overall feedback orientation and academic emotions.

Consistent with the proposed model, we hypothesized that:

*H1*: Overall feedback orientation would be positively associated with agentic engagement.

*H2*: Agentic engagement would be positively associated with academic emotions after overall feedback orientation was taken into account.

*H3*: Overall feedback orientation would show a positive indirect association with academic emotions through agentic engagement.

The model also estimated the direct association between feedback orientation and academic emotions after agentic engagement was included. All hypotheses concern associations within a cross-sectional SEM and do not establish temporal ordering or causal mediation.

## Methods

### Participants

Participants were recruited from a secondary vocational school in Henan Province, mainland China, using purposive convenience sampling. The final sample comprised 452 students. Most participants were female (98.9%), reflecting the gender composition of the programs from which the sample was drawn. Participants were enrolled in Preschool Education (63.9%), Early Childhood Care (31.2%), or Art Design and Production (4.9%). Their ages ranged from 16 to 21 years (*M* = 17.66, *SD* = 1.20).

The sample was concentrated within programs preparing students for preschool education and early-childhood-care occupations at one institution. Consequently, the findings should be interpreted in relation to this specific institutional and program context. Gender- and program-based comparisons were not undertaken because only five male students participated and the program groups were substantially unequal in size (see Table 1).

**Table 1:** Demographic characteristics of participants (*N* = 452).

Characteristic	*n*	%
Gender		
Female	447	98.9
Male	5	1.1
Teacher-training status		
Teacher-training students	432	95.6
Non-teacher-training students	20	4.4
Program		
Preschool Education	289	63.9
Early Childhood Care	141	31.2
Art Design and Production	22	4.9

### Measures

#### Short-form feedback orientation scale

Feedback orientation was assessed using a 12-item short form of the Feedback Orientation Scale. The original 20-item scale was developed by Linderbaum and Levy (2010) to assess feedback orientation in organizational settings. It was subsequently translated into Chinese and adapted for educational contexts by Yang et al. (2014).

The short form measured four dimensions of feedback orientation: feedback utility, feedback self-efficacy, feedback social awareness, and feedback accountability. Each dimension was represented by three items. Participants indicated their agreement with each item on a 4-point Likert-type scale. Higher scores represented stronger feedback orientation. Previous studies have supported its multidimensional internal structure across secondary and higher-education samples (Yang et al., 2023a; Yang et al., 2023b). Confirmatory factor analysis was conducted to evaluate the factorial validity of the 12-item measure in the present secondary vocational education sample.

#### Agentic engagement scale

Agentic engagement was assessed using the five-item agentic engagement subscale from the student engagement measure developed by Reeve and Tseng (2011). The broader measure assesses agentic, behavioral, emotional, and cognitive engagement; only the agentic engagement subscale was used because the present study focused on students’ proactive contributions to feedback-related learning processes.

Participants responded to the items using a 4-point Likert-type scale. A sample item was “I express my feelings and reactions to the feedback.” Higher scores indicated greater agentic engagement in feedback processes.

#### Academic emotions scale

Academic emotions were assessed using an eight-item measure developed by Yang (2018) to examine students’ learning-related achievement emotions. Academic emotions refer to emotional experiences associated with learning activities and academic outcomes and may include pleasant and unpleasant emotions (Bordbar, 2019). A sample item was “After studying, I feel happy with my progress, which encourages me to continue learning.” Participants responded using a 4-point Likert-type scale. Higher scores indicated more positive academic emotions. In the present study, the scale demonstrated high internal consistency, with Cronbach’s α = 0.946.

### Procedure

Ethical approval was obtained from the relevant institutional ethics committee before data collection. The questionnaire was administered online through Wenjuanxing, an online survey platform widely used in mainland China. Before completing the questionnaire, participants were informed of the purpose of the study, the voluntary nature of participation, the confidentiality of their responses, and their right to withdraw at any time without penalty. They were also informed that participation involved no anticipated risks and that no compensation would be provided. Students who agreed to participate accessed the survey through a hyperlink. Responses were recorded anonymously and stored on the survey platform before being exported for statistical analysis.

### Statistical analysis

This study used SPSS, Mplus, and R (version 4.5.0; R Core Team, 2025) to process the data. SPSS was for measuring descriptive statistics, skewness, and reliability. R and Mplus were applied for examining competing models. The R environment (e.g., *lavvan psych* packages) was utilized for confirmatory factor analyses and structural equation modeling (SEM) analyses (Revelle and Revelle, 2015).

Descriptive statistics were calculated to characterize the distributions of the study variables. Skewness and kurtosis were inspected as indicators of univariate normality. Internal consistency was examined using Cronbach’s α, and Pearson correlations were calculated among the four feedback-orientation dimensions, agentic engagement, and academic emotions. These analyses provided an initial description of the data and associations before latent-variable modeling (Kelloway, 2014; Wang and Wang, 2019).

Confirmatory factor analysis (CFA) compared four theoretically plausible representations of the 12-item short-form Feedback Orientation Scale: a one-factor model, a correlated four-factor model, a second-order model, and a bifactor model. Model evaluation considered global fit together with factor loadings, composite reliability, average variance extracted, factor correlations, and the interpretability of the proposed dimensions. This cumulative approach was appropriate because feedback utility, self-efficacy, social awareness, and accountability are theoretically distinguishable while also expected to share common variance.

Prior to measuring structural model, we examined the measurement structure of academic emotions. Because the six items reflects two distinct types of positive emotions (e.g., enjoyment with 3 items, hope with 3 items), we further compared a one-factor model against second-order factor model. The second-order model (*χ*^2^ = 9.99, df = 8, CFI = 0.997, TLI = 0.995, RMSEA = 0.047, SRMR = 0.015) fit substantially better than the one-factor model (*χ*^2^ = 29.22, df = 9, CFI = 0.975, TLI = 0.959, RMSEA = 0.137, SRMR = 0.025), supporting enjoyment and hope as a second-order factor (*r* = 0.94). Therefore, we specified academic emotions as second-order factor. Besides, agentic engagement was specified as a unidimensional latent factor (CFI = 0.967, SRMR = 0.028), consistent with its conceptual definition. Feedback orientation was defined as a second-order factor with four dimensions (e.g., FBUT, FBSE, FBSO, FBAT).

The SEM was then used to estimate the hypothesized concurrent direct and indirect associations among overall feedback orientation, agentic engagement, and academic emotions (e.g., enjoyment, hope). Feedback orientation was modeled as a second-order latent construct; agentic engagement was specified as the intermediary variable, and academic emotions as the outcome.

Model fit was evaluated using *χ*^2^, *χ*^2^/df, CFI, TLI, RMSEA with its 90% confidence interval, and SRMR. As descriptive benchmarks, CFI and TLI values of 0.90 or above are commonly regarded as indicating acceptable fit and values approaching 0.95 as favorable; SRMR values below 0.08 and RMSEA values at or below 0.08 are commonly treated as evidence of reasonable approximation (Hu and Bentler, 1999; MacCallum et al., 1996).

Because the items exhibited negative skewness, we used robust maximum likelihood estimation (MLR) with the Satorra-Bentler correction (Satorra and Bentler, 1994), which corrects the chi-square and standard error for non-normality. The indirect association was tested using 5,000 bias-corrected bootstrap resamples. An indirect association was regarded as statistically supported when its 95% confidence interval excluded zero. Standardized and unstandardized estimates were clearly distinguished in reporting. As all constructs were measured through self-report in the same online survey, SEM estimates were interpreted as concurrent associations and not as evidence of temporal or causal mediation.

Besides, the linear research procedure of the study includes design type, target population, sampling method and sample size, instrumentation and data collection (also including reliability and validity tools), and data analysis and interpretation, so that the study can be replicated.

## Results

### Preliminary and correlational analyses

Univariate normality was examined before the measurement and structural models were estimated. As reported in Table 2, skewness values ranged from −1.09 to −0.38, and kurtosis values ranged from −1.09 to 0.938. Table 3 presents item-level correlations and descriptive statistics. The four feedback-orientation dimensions were positively correlated with agentic engagement and academic emotions, and agentic engagement was positively correlated with academic emotions; these are concurrent bivariate associations and do not establish directionality.

**Table 2:** Variable-level correlations matrix, CR, and AVE of the four factors of FO, AE, and ACEM.

Factors	M	SD	Skewness	Kurtosis	1	2	3	4	5	6	7	8	CR	AVE	a
FBUT	3.29	0.73	−0.78	0.066	–								0.931	0.820	0.921
FBSE	3.37	0.70	−0.91	0.356	0.914***	–							0.966	0.904	0.965
FBSO	3.35	0.72	−0.89	0.219	0.908***	0.930***	–						0.954	0.873	0.953
FBAT	3.32	0.73	−0.83	0.135	0.895***	0.901***	0.940***	–					0.948	0.860	0.943
AE	3.12	0.81	−0.38	−1.09	0.613***	0.617***	0.627***	0.623***	–				0.958	0.821	0.956
ACEM	3.57	0.54	−1.05	0.864	0.510***	0.540***	0.523***	0.517***	0.598***	–			0.961	0.802	0.960
Enjoyment	3.57	0.54	−1.04	0.778	0.486***	0.508***	0.492***	0.490***	0.580***	0.969***	–		0.917	0.787	0.915
Hope	3.56	0.57	−1.09	0.938	0.504***	0.539***	0.522***	0.514***	0.581***	0.973***	0.885***	–	0.955	0.877	0.955

*p < 0.05, **p < 0.01. feedback utility = FBUT; feedback self-efficacy = FBSE; feedback social awareness = FBSO; feedback accountability = FBAT; agentic engagement = AE; a = Cronbach’s a; enjoyment and hope are two components of positive academic emotions (ACEM).

**Table 3:** Item-level correlation coefficient matrix of FO (four dimensions; 12 items), AE (5 items), and ACEM (8 items), and descriptive statistics.

	1	2	3	4	5	6	7	8	9	10	11	12	13	14	15	16	17	18	19	20	21	22	23
FBUT1	–																						
FBUT2	0.751^**^	–																					
FBUT3	0.729^**^	0.937^**^	–																				
FBSE1	0.725^**^	0.909^**^	0.892^**^	–																			
FBSE2	0.728^**^	0.906^**^	0.885^**^	0.913^**^	–																		
FBSE3	0.688^**^	0.869^**^	0.854^**^	0.880^**^	0.917^**^	–																	
FBSO1	0.713^**^	0.841^**^	0.853^**^	0.871^**^	0.876^**^	0.909^**^	–																
FBSO2	0.733^**^	0.837^**^	0.829^**^	0.832^**^	0.817^**^	0.836^**^	0.833^**^	–															
FBSO3	0.744^**^	0.885^**^	0.872^**^	0.864^**^	0.864^**^	0.877^**^	0.891^**^	0.891^**^	–														
FBAT1	0.745^**^	0.867^**^	0.839^**^	0.881^**^	0.869^**^	0.881^**^	0.897^**^	0.890^**^	0.917^**^	–													
FBAT2	0.717^**^	0.862^**^	0.831^**^	0.874^**^	0.861^**^	0.877^**^	0.882^**^	0.870^**^	0.894^**^	0.941^**^	–												
FBAT3	0.743^**^	0.778^**^	0.770^**^	0.775^**^	0.731^**^	0.725^**^	0.748^**^	0.802^**^	0.806^**^	0.810^**^	0.811^**^	–											
AE1	0.451^**^	0.487^**^	0.478^**^	0.459^**^	0.463^**^	0.486^**^	0.505^**^	0.478^**^	0.507^**^	0.500^**^	0.473^**^	0.501^**^	–										
AE2	0.457^**^	0.516^**^	0.521^**^	0.505^**^	0.498^**^	0.527^**^	0.540^**^	0.530^**^	0.521^**^	0.515^**^	0.523^**^	0.505^**^	0.797^**^	–									
AE3	0.498^**^	0.570^**^	0.565^**^	0.563^**^	0.554^**^	0.590^**^	0.575^**^	0.564^**^	0.567^**^	0.570^**^	0.571^**^	0.524^**^	0.752^**^	0.866^**^	–								
AE4	0.522^**^	0.574^**^	0.562^**^	0.584^**^	0.575^**^	0.607^**^	0.602^**^	0.546^**^	0.557^**^	0.594^**^	0.585^**^	0.545^**^	0.734^**^	0.831^**^	0.897^**^	–							
AE5	0.529^**^	0.618^**^	0.612^**^	0.638^**^	0.622^**^	0.648^**^	0.648^**^	0.593^**^	0.604^**^	0.638^**^	0.626^**^	0.564^**^	0.711^**^	0.816^**^	0.879^**^	0.918^**^	–						
ACEM1_JO	0.392^**^	0.471^**^	0.465^**^	0.460^**^	0.484^**^	0.512^**^	0.475^**^	0.445^**^	0.461^**^	0.473^**^	0.457^**^	0.388^**^	0.394^**^	0.454^**^	0.520^**^	0.530^**^	0.542^**^	–					
ACEM2_JO	0.332^**^	0.435^**^	0.416^**^	0.417^**^	0.428^**^	0.441^**^	0.417^**^	0.399^**^	0.404^**^	0.440^**^	0.443^**^	0.380^**^	0.409^**^	0.475^**^	0.516^**^	0.548^**^	0.549^**^	0.789^**^	–				
ACEM3_JO	0.371^**^	0.467^**^	0.437^**^	0.443^**^	0.444^**^	0.463^**^	0.438^**^	0.415^**^	0.457^**^	0.461^**^	0.455^**^	0.392^**^	0.426^**^	0.479^**^	0.520^**^	0.556^**^	0.562^**^	0.728^**^	0.833^**^	–			
ACEM4_HO	0.375^**^	0.482^**^	0.486^**^	0.479^**^	0.493^**^	0.514^**^	0.494^**^	0.451^**^	0.481^**^	0.477^**^	0.467^**^	0.430^**^	0.411^**^	0.467^**^	0.522^**^	0.562^**^	0.568^**^	0.789^**^	0.790^**^	0.793^**^	–		
ACEM5_HO	0.363^**^	0.485^**^	0.479^**^	0.492^**^	0.495^**^	0.516^**^	0.502^**^	0.458^**^	0.484^**^	0.484^**^	0.489^**^	0.427^**^	0.416^**^	0.471^**^	0.532^**^	0.560^**^	0.580^**^	0.791^**^	0.814^**^	0.824^**^	0.902^**^	–	
ACEM6_HO	0.415^**^	0.499^**^	0.487^**^	0.492^**^	0.501^**^	0.514^**^	0.492^**^	0.463^**^	0.483^**^	0.499^**^	0.505^**^	0.437^**^	0.415^**^	0.501^**^	0.557^**^	0.597^**^	0.595^**^	0.748^**^	0.759^**^	0.756^**^	0.867^**^	0.861^**^	–
M	3.21	3.34	3.33	3.35	3.37	3.39	3.36	3.34	3.35	3.36	3.36	3.25	2.99	3.07	3.17	3.18	3.20	3.53	3.59	3.60	3.56	3.58	3.54
SD	0.856	0.738	0.757	0.743	0.731	0.707	0.753	0.756	0.754	0.741	0.730	0.831	0.957	0.904	0.849	0.839	0.850	0.597	0.571	0.570	0.602	0.573	0.618
Skewness	−0.839	−0.870	−0.944	−0.931	−0.969	−0.901	−0.948	−0.966	−0.977	−0.915	−0.917	−0.943	−0.429	−0.443	−0.503	−0.534	−0.627	−0.991	−1.194	−1.298	−1.160	−1.213	−1.180
Kurtosis	−0.091	0.154	0.377	0.299	0.476	0.236	0.233	0.422	0.461	0.188	0.297	0.246	−0.993	−0.988	−0.983	−0.896	−0.713	0.635	1.220	1.865	0.934	1.630	1.133

**p < 0.01; *p < 0.05 JO = enjoyment. HO = hope; enjoyment and hope are two components of positive academic emotions.

The 12-item Feedback Orientation Scale produced Cronbach’s α = 0.977. This high coefficient was interpreted together with the content of the four dimensions, the competing CFA models, and prior psychometric studies of the short form (e.g., Yang et al., 2023a; Yang et al., 2023b) rather than as stand-alone evidence. Composite reliability ranged from 0.932 to 0.966 and average variance extracted ranged from 0.707 to 0.904, supporting internal consistency and convergent validity in this sample. The strong relations among the four dimensions are consistent with feedback orientation as a broad disposition expressed through closely related facets.

Discriminant validity among the three broader constructs was further examined using the Heterotrait–Monotrait Ratio of Correlations (HTMT; Henseler et al., 2015). HTMT values were 0.661 for feedback orientation–agentic engagement, 0.556 for feedback orientation–positive academic emotions, and 0.626 for agentic engagement–positive academic emotions, all below the conservative 0.85 criterion and supporting their empirical differentiation. Detailed item-level calculations are provided in Appendix Table A1.

### Correlational analysis

Tables 2, 3 present the descriptive statistics and bivariate correlations among the four dimensions of feedback orientation, agentic engagement, and academic emotions. All four feedback-orientation dimensions were positively and significantly correlated with agentic engagement. These findings are consistent with prior evidence that students’ orientations toward feedback are associated with their engagement in feedback-related activities (Yang and Yang, 2018). The four dimensions were also positively correlated with academic emotions. This pattern is broadly consistent with research suggesting that students’ interpretations of feedback are related to their emotional responses to feedback experiences (Yang et al., 2023c). Agentic engagement was also positively correlated with academic emotions.

### Measurement models of the short-form feedback orientation scale and academic emotions

Confirmatory factor analyses were conducted to examine the measurement structures of feedback orientation and academic emotions. For the 12-item short-form Feedback Orientation Scale, four alternative models were compared: a one-factor model, a correlated four-factor model, a second-order model, and a bifactor model. Table 4 presents the corresponding fit indices.

**Table 4:** Fit indices and results of CFA for each measurement model.

Models	*X^2^*	*df*	RMSEA (90% CI)	CFI	TLI	SRMR	AIC	BIC
M1	177.374*	54	0.071 (0.060, 0.083)	0.926	0.910	0.023	3980.078	4128.170
M2	100.374*	48	0.049 (0.036, 0.063)	0.969	0.957	0.024	3567.822	3740.596
M3	111.948*	50	0.052 (0.039, 0.065)	0.963	0.951	0.024	3652.347	3816.894
M4	92.451*	42	0.052 (0.037, 0.066)	0.970	0.953	0.020	3577.789	3775.245

M1 = one-factor model; M2 = correlated four-factor model; M3 = second-order model; M4 = bifactor model. AIC = Akaike information criterion; BIC = Bayesian information criterion; CFI = comparative fit index; RMSEA = root mean square error of approximation; SRMR = standardized root mean square residual; TLI = Tucker–Lewis index. *p < 0.01.

As shown in Table 4, the one-factor model provided the weakest representation of feedback orientation, indicating that the 12 items should not be treated as a single undifferentiated construct. In contrast, the correlated four-factor, second-order, and bifactor models all showed good fit, supporting the multidimensional structure of feedback orientation while also indicating substantial commonality across its four dimensions. Although the correlated four-factor model showed slightly better information criteria and the bifactor model comparable fit, these differences were small. Given the theoretical conceptualization of feedback orientation as an overarching construct comprising utility, self-efficacy, social awareness, and accountability, and the strong associations among these dimensions, the second-order model was retained for the subsequent SEM. This specification preserves the theoretically meaningful lower-order dimensions while allowing feedback orientation to be examined parsimoniously as a broader latent construct.

For academic emotions, two alternative measurement models were examined. The one-factor model, in which all six items loaded on a single factor, showed mixed fit, *χ*^2^(9) = 29.22, *χ*^2^/df = 3.25, CFI = 0.975, TLI = 0.959, RMSEA = 0.137, 90% CI [0.084, 0.194], and SRMR = 0.025. In contrast, the correlated two-factor model comprising enjoyment (three items) and hope (three items) showed good fit, *χ*^2^(8) = 9.99, *χ*^2^/df = 1.25, CFI = 0.997, TLI = 0.995, RMSEA = 0.047, 90% CI [0.000, 0.126], and SRMR = 0.015. The latent correlation between enjoyment and hope was high (*r* = 0.943), indicating substantial common variance while retaining the distinction between the two emotion dimensions. Given this strong commonality, enjoyment and hope were represented as first-order dimensions of a broader academic-emotions construct in the subsequent SEM. Accordingly, the structural model specified both feedback orientation and academic emotions as higher-order latent constructs to examine the associations among feedback orientation, agentic engagement, and academic emotions.

### Structural equation model

Structural equation modeling estimated the concurrent associations among overall feedback orientation, agentic engagement, and academic emotions while accounting for measurement error (see Figure 1 and Table 5). Informed by the preceding measurement analyses, feedback orientation was specified as a higher-order latent construct represented by feedback utility, feedback self-efficacy, feedback social awareness, and feedback accountability. Academic emotions were likewise represented at the broader construct level through the highly related enjoyment and hope dimensions, while agentic engagement was specified as the intermediary variable. This model enabled estimation of the direct association between feedback orientation and academic emotions and the indirect association statistically accounted for by agentic engagement. The specification retained the theoretically distinguishable lower-order dimensions of feedback orientation and academic emotions while modeling their shared variance at the broader construct level. Given the cross-sectional design, these estimates represent concurrent associations and do not establish temporal ordering or causal mediation (Kelloway, 2014; Wang and Wang, 2019).

**Figure 1:**
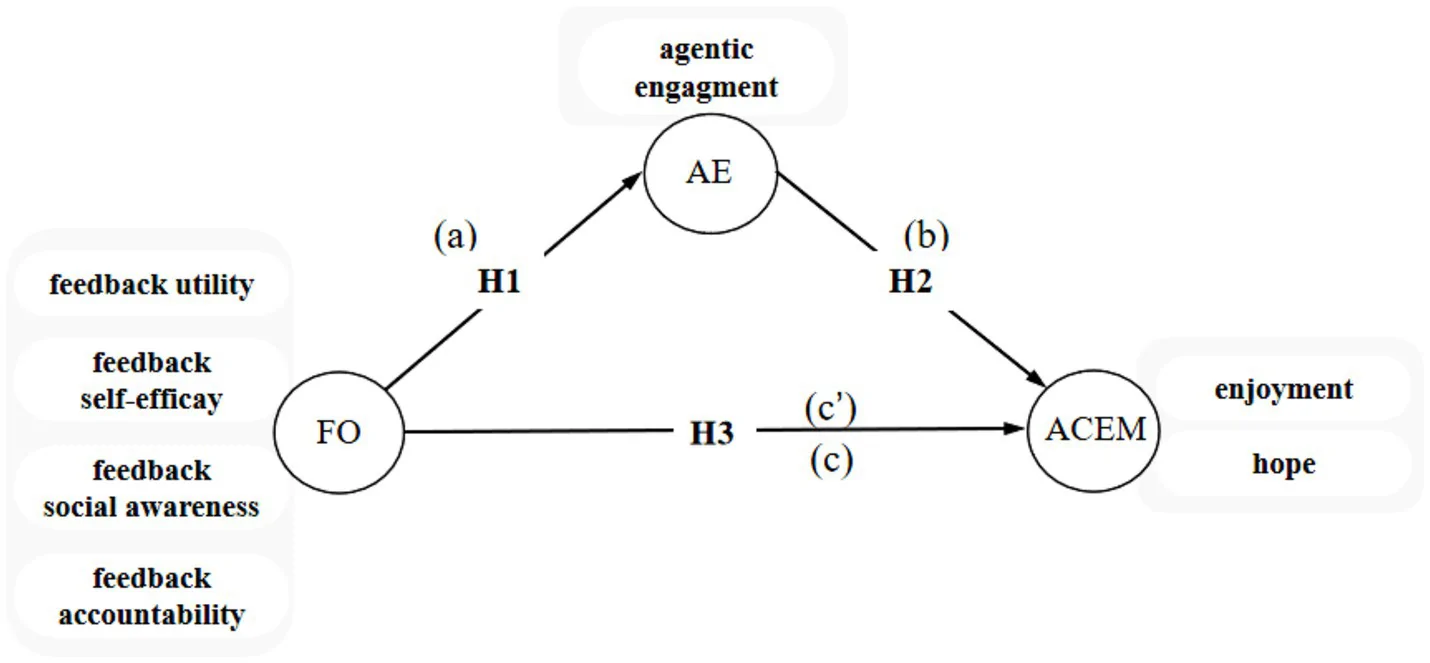
Cross-sectional structural model linking feedback orientation, agentic engagement, and academic emotions. Feedback orientation and academic emotions are represented as higher-order latent constructs. Agentic engagement is specified as the intermediary variable linking feedback orientation with academic emotions. The model estimates both the direct association between feedback orientation and academic emotions and the indirect association statistically accounted for by agentic engagement. Arrows indicate the specified structural associations and should not be interpreted as establishing temporal ordering or causal effects.

**Table 5:** Structural associations, indirect association, and explained variance in the SEM (*N* = 452).

Association	*B*	SE	*β*	*z*	*p*	95% CI	Explained variance (*R*^2^)
Direct associations							
Feedback orientation → Agentic engagement	0.782	0.064	**0.662**	12.227	<0.001	[0.659, 0.908]	0.438
Agentic engagement → Academic emotions	0.319	0.049	**0.482**	6.534	<0.001	[0.226, 0.420]	0.445
Feedback orientation → Academic emotions	0.189	0.055	**0.241**	3.421	<0.001	[0.081, 0.300]	
Indirect association							
Feedback orientation → Agentic engagement → Academic emotions	0.249	0.038	**0.319**	6.582	<0.001	[0.178, 0.330]	
Total association							
Feedback orientation → Academic emotions	0.438	0.047	**0.560**	9.237	<0.001	[0.346, 0.537]	

B = unstandardized coefficient; SE = standard error; β = standardized coefficient (STDYX); z = z-statistic; CI = 95% bias-corrected bootstrap confidence interval based on 5,000 resamples; *R*^2^ = proportion of explained variance. *p* values indicate statistically significant.

### Indirect-association analysis

The structural model demonstrated good overall fit, *χ^2^*(221) = 433.56, *p* < 0.001, *χ^2^/df* = 1.96, RMSEA = 0.067, CFI = 0.971, TLI = 0.966, and SRMR = 0.028. Taken together, the fit indices indicated satisfactory correspondence between the specified model and the observed data. The model represented feedback orientation as a higher-order latent construct comprising feedback utility, feedback self-efficacy, feedback social awareness, and feedback accountability, and academic emotions as a higher-order construct represented by enjoyment and hope.

Feedback orientation was positively associated with agentic engagement, *β* = 0.662, *p* < 0.001, supporting H1. Agentic engagement, in turn, was positively associated with academic emotions while accounting for feedback orientation, *β* = 0.482, *p* < 0.001, supporting H2. Feedback orientation also retained a positive direct association with academic emotions, *β* = 0.241, *p* = 0.001.

The indirect association between feedback orientation and academic emotions through agentic engagement was also positive and statistically significant. The unstandardized indirect association was *B* = 0.249, *SE* = 0.038, 95% CI [0.175, 0.324], *p* < 0.001, and the corresponding standardized indirect association was *β* = 0.319. The total standardized association between feedback orientation and academic emotions was *β* = 0.560, indicating that approximately 56% of the total association was statistically accounted for through agentic engagement. These findings supported H3 at the level of a statistically significant indirect association.

## Discussion

This study examined concurrent associations among feedback orientation, agentic engagement, and academic emotions and evaluated the structure of a 12-item short-form Feedback Orientation Scale. The CFA results supported a correlated four-factor representation and were also consistent with substantial common variance across the four dimensions. Overall feedback orientation was positively associated with agentic engagement and academic emotions, and the specified SEM contained a statistically supported indirect association through agentic engagement. These findings are interpreted within the boundaries of common-source measurement and the cross-sectional design.

The study extends descriptive evidence to a narrowly defined vocational context dominated by preschool education and early-childhood-care programs. In such programs, feedback is often closely linked to repeated performance of observable professional tasks. A favorable feedback orientation may help students treat repeated correction as information for improvement rather than only as external evaluation. However, the study did not compare vocational and general education, measure instructional repetition or hierarchy, or assess workplace performance; claims about distinctive vocational mechanisms therefore remain theoretical. The interpretation is consistent with research describing the task-centered and vocational-becoming demands of secondary vocational learning (Bochkareva et al., 2020; Johansson, 2020; Smolyakova, 2021).

### Feedback orientation and academic emotions

The positive direct association between feedback orientation and academic emotions was consistent with previous research linking feedback-related perceptions and achievement emotions (Frondozo and Yang, 2023; Pekrun et al., 2014; Yang et al., 2023a). Students who perceived feedback as valuable, felt capable of interpreting and using it, recognized its interpersonal significance, and accepted responsibility for responding to it tended to report more positive academic emotions.

This association can be interpreted through students’ appraisals of feedback. Feedback perceived as useful and manageable may communicate that improvement is possible, whereas feedback interpreted as threatening, irrelevant, or difficult to use may be accompanied by less adaptive emotional experiences. Nevertheless, the present study assessed feedback orientation and academic emotions concurrently. It cannot determine whether feedback orientation shaped students’ emotions, whether emotional experiences influenced how students perceived feedback, or whether both were influenced by unmeasured characteristics of the learning environment. This appraisal-based interpretation is consistent with evidence that anticipated and experienced feedback are related to achievement emotions (Pekrun et al., 2014; Yang et al., 2023c).

The study measured academic emotions as an overall construct. The results therefore do not establish that feedback orientation was related specifically to greater joy or hope or to lower anxiety or boredom unless these individual emotions were separately modeled. Claims concerning particular positive and negative emotions should be reserved for analyses that distinguish their respective dimensions.

### Indirect association involving agentic engagement

The findings highlight agentic engagement as a potentially important positive contributor linking students’ feedback orientation with their academic emotions. Students with stronger feedback orientation tended to engage more agentically, and such engagement was associated with more positive academic emotions. Although the cross-sectional design precludes conclusions about temporal or causal ordering, this pattern identifies agentic engagement as a meaningful component of students’ positive feedback experiences.

Conceptually, the findings distinguish being positively oriented toward feedback from actively contributing to feedback processes. Feedback orientation reflects students’ readiness and resources for engaging with feedback, whereas agentic engagement captures their constructive contribution through questioning, clarification, expressing needs, and seeking information (Reeve, 2013; Reeve and Jang, 2022). Thus, positive feedback orientation may be particularly consequential when students can actively participate in and shape feedback interactions, consistent with proactive feedback recipience (Winstone et al., 2017).

Agentic engagement did not, however, statistically account for the entire association between feedback orientation and academic emotions. Other processes, including competence appraisal, emotion regulation, feedback credibility, and relational quality, may also contribute. Future longitudinal and multi-method research could examine how these processes operate together and whether positive feedback orientation is translated into more adaptive emotional experiences through students’ active contribution to feedback processes.

### Interpretation through self-determination theory

The findings are compatible with, but do not directly test, the SDT-informed articulation advanced by Yang and Yang (2018). Nevertheless, the present study did not directly measure autonomy, competence, relatedness, or their frustration. It therefore cannot establish that the observed relationships occurred because basic psychological needs were satisfied. Claims that agentic engagement represents the satisfaction of these needs should be moderated. A more defensible conclusion is that the results are consistent with an SDT-informed account in which feedback-related dispositions and proactive participation are associated with students’ emotional experiences. This qualification follows SDT’s distinction between contextual support, basic-need experience, and the regulatory quality of behavior (Ryan and Deci, 2000, 2017).

Previous research suggests that agentic engagement allows students to voice their preferences and participate in shaping the instructional information and support available to them (Patall, 2024). Proactive classroom participation may also be associated with autonomy, opportunities to exchange feedback, and stronger interpersonal connection (Kusurkar et al., 2011; Xie and Chew, 2025). The current findings add evidence that agentic engagement is positively associated with academic emotions in feedback-related learning. They do not, however, demonstrate that students’ proactive actions necessarily produced need satisfaction or emotional change.

The theoretical contribution is therefore interactional rather than linear: students may bring feedback-related dispositions and attempt to shape feedback dialog, but the emotional significance of those actions may depend on whether the instructional environment acknowledges and responds to them. Future research should test this person–context proposition by measuring teacher responsiveness, need satisfaction and frustration, autonomous and controlled reasons for acting on feedback, and discrete academic emotions over time.

This context-sensitive interpretation also addresses the particular tension of vocational learning: agentic engagement is unlikely to be beneficial simply because students act proactively. In a highly structured environment, the consequences of asking questions, seeking clarification, or proposing alternatives may depend on whether teachers and task structures permit those contributions to influence subsequent learning. SDT-related work on novelty suggests that opportunities for new, self-endorsed experience can be relevant to well-being in structured educational settings (González-Cutre et al., 2016; Trigueros and García-Mas, 2025), while Franco et al. (2026) points to the potential importance of self-regulatory resources for adaptive study processes. Accordingly, feedback orientation and agentic engagement should be understood as resources whose emotional significance is conditioned by the responsiveness of the learning environment and students’ capacity to regulate their own learning. The present data did not measure novelty, mindfulness, regulatory quality, or teacher responsiveness, so these possibilities remain directions for future testing rather than explanations established by the current model.

### Practical implications

Within the preschool-education and early-childhood-care programs represented in this study, the findings suggest that feedback may be most productively framed as information that helps students interpret performance standards and identify a feasible next action, rather than as correction alone. For practical tasks involving observable procedures or performance criteria, teachers could consider making the relevant standard and the gap between current and expected performance explicit, inviting students to identify a possible revision for a subsequent attempt, and creating space for clarification where feedback is unclear. These possibilities are consistent with feedback literature emphasizing learners’ active interpretation and use of feedback (Ajjawi et al., 2022; Carless, 2022; Winstone et al., 2017).

The observed association between feedback orientation and agentic engagement also suggests that students’ questions, uncertainty, and requests for explanation may be understood as potentially productive contributions to the feedback process. In settings involving externally specified procedures, responsive feedback dialog may help students engage more actively with feedback rather than simply follow correction. This possibility is consistent with autonomy- and competence-supportive teaching, which has been associated with more favorable student experiences in the wider SDT literature (Howard et al., 2025; Reeve and Jang, 2022). These implications are theoretically informed and suggestive; they are not intervention effects established by the present cross-sectional study.

### Limitations and directions for future research

The findings provide evidence concerning the measurement and concurrent relations of feedback orientation in an underexamined secondary vocational context. Their interpretation is nevertheless bounded by several design features. The cross-sectional design cannot determine whether feedback orientation precedes agentic engagement and academic emotions, or whether these variables influence one another over time. Longitudinal, intensive longitudinal, and intervention studies could examine developmental ordering, reciprocal relations, and within-student change, while testing whether feedback-oriented dispositions are translated into feedback-related action over time (Carless, 2022; Winstone et al., 2017).

All variables were measured through students’ self-reports in one survey. The observed associations therefore represent students’ reported perceptions and experiences within a common measurement occasion. Subsequent research could extend this evidence through temporally separated measures, theoretically justified method controls, teacher reports or observations of agentic engagement, records of feedback use, and repeated assessments of emotions. Such designs would better capture feedback as an enacted, relational process rather than solely as a reported disposition or experience (Ajjawi et al., 2022; Reeve and Jang, 2022).

The short-form scale showed very high overall internal consistency in this program-specific sample. This result was interpreted alongside the competing CFA models, composite reliability, average variance extracted, item content, and previous psychometric evidence (Linderbaum and Levy, 2010; Yang et al., 2014). The results support modeling an overall feedback-orientation factor while retaining its four theoretically meaningful dimensions. Replication across more diverse vocational programs could further examine omega reliability, bifactor indices, cross-loadings, and the relative usefulness of overall and dimension-specific scores.

When academic emotions were represented as a higher-order construct comprising enjoyment and hope, the structural model showed good overall fit, with favorable CFI, TLI, RMSEA, and SRMR values. This improved fit strengthens confidence that the proposed model provides an adequate representation of the observed associations among feedback orientation, agentic engagement, and academic emotions. At the same time, the high shared variance between enjoyment and hope suggests that these dimensions are meaningfully related within a broader positive academic-emotion construct, while remaining theoretically distinguishable at the lower-order level. The findings should nevertheless be interpreted within the boundaries of the present sample. Because participants were drawn from a single secondary vocational school and were concentrated in preschool education and early-childhood-care programs, replication across more diverse school, program, and student populations is needed to evaluate the generalizability and stability of the model.

Teacher responsiveness, students’ reasons for acting on feedback, and discrete positive and negative emotions were not assessed. Future research could examine whether students’ agentic contributions receive autonomy-, competence-, and relatedness-supportive responses, and whether these responses condition the emotional consequences of feedback engagement (Howard et al., 2025; Ryan and Deci, 2017). It could also distinguish self-endorsed from controlled reasons for feedback accountability and assess discrete emotions, because feedback appraisals may relate differently to enjoyment, hope, anxiety, shame, or frustration (Pekrun et al., 2014; Yang et al., 2023c).

## Conclusion

This study extends feedback-orientation research to secondary vocational education by supporting feedback orientation as a multidimensional but strongly integrated construct and demonstrating its concurrent associations with agentic engagement and positive academic emotions. Agentic engagement statistically accounted for part of the association between feedback orientation and positive academic emotions, suggesting that how students orient toward feedback is meaningfully connected with how actively they participate in learning and how they experience it emotionally. These findings position feedback orientation as a relevant learner resource in vocational education while underscoring the need for longitudinal and multi-method research to establish temporal processes and generalizability.

## Data Availability

The raw data supporting the conclusions of this article will be made available by the authors, without undue reservation.
