# Supplementary material for: From feedback orientation to academic emotions: the indirect association through agentic engagement among secondary vocational students
Source: Front Psychol. 2026 Oct 19;17:1959575. doi: 10.3389/fpsyg.2026.1959575 (PMC13724406; doi:10.3389/fpsyg.2026.1959575)
Supplement: Supplementary file 1 [file Supplementary_file_1.docx]

**Appendices**

**Table A1.**

*Item-Level HTMT Calculations for Feedback Orientation, Agentic Engagement, and Positive Academic Emotions*

| Construct comparison | Mean cross-construct *r* | Mean within first construct *r* | Mean within second construct *r* | HTMT |
| --- | --- | --- | --- | --- |
| FO – Agentic Engagement | 0.548 | FO = 0.836 | Agentic Engagement = 0.820 | **0.661** |
| FO – Positive Academic Emotions | 0.455 | FO = 0.836 | Positive Academic Emotions = 0.803 | **0.556** |
| Agentic Engagement – Positive Academic Emotions | 0.508 | Agentic Engagement = 0.820 | Positive Academic Emotions = 0.803 | **0.626** |

**Figure A1.** The one-factor model of FO.

**Figure A2.** The four-factor model of sf-FOS.

**Figure A3.** The second-order factor of FO.

**Figure A4.** The bi-factor model of FO.
